# Health of New Orleans

**Published:** 1844-11-16

**Authors:** 


### HEALTH OF NEW ORLEANS.

The health of New Orleans was perhaps never known to be better than from the beginning of this year up to the present time. No epidemic whatever has prevailed, and the most extensive practitioners of the place unite in the testimony that they have never had less to do during the same period.

The summer has been one of the hottest ever experienced; with frequent showers during July and August, as will be seen by the abstract of our meteorological tables. Thus it would appear we have had a large share of *two*, of what have generally been considered the most *essential agents* in the production of the remote cause of summer and autumnal diseases, i. e. *heat* and *moisture*. As to the other ingredients, viz. *dead vegetable and animal matter*, one would suppose there was never any deficiency about such a place as New Orleans. Well, we have here all the *hypothetical elements* of *hypothetical malaria*—but where are the much dreaded consequences? We will go on with our statement of facts, and our readers may draw their own conclusions. The Mississippi which runs along the border of our city for about three miles, has probably not been higher within *fifty years* past. Its waters being considerably above the level of the city, were permitted by means of culverts to pass into the gutters, and thus to flow along the principal streets in continued streams to the swamp in the rear. These lively streams, to the width of at least a mile along the heart of the city, have continued to flow from May to about the 10th of September, when their source was cut off by the falling of the river. The descent from the river to the swamp, is about 4 or 5 feet; lessening as you recede till the waters in the gutters at the back part of the city, (a distance of a half, to three quarters of a mile), are almost stagnant. The scavengers usually draw out the thick, muddy contents, with hoes, and after drying, it is carted off. The supply of fresh water this season must have had a salutary influence upon these *filthy sewers*.

Since the decline of the river, an immense *batture* along the whole extent of the city has been exposed to the rays of an autumnal sun, but little mitigated by clouds or rain, for four or five weeks past. The effluvia from this batture is quite offensive, both in the morning and evening.

Such is the state of the case; and under such influences, New Orleans has been, and continues to be, one of the healthiest cities in the world.

By reference to our Hospital Reports, it will be seen the admissions into the Charity Hospital have been unusually large; but the cause of this is explained in our last number, and need not be repeated here. It is gratifying to witness the decided amelioration in the mortality of that Institution. The monthly statements of the other Hospitals afford a better index to the state of health.—*N. O. Medical Journal.*

